# Fast and efficient microfluidic cell filter for isolation of circulating tumor cells from unprocessed whole blood of colorectal cancer patients

**DOI:** 10.1038/s41598-019-44401-1

**Published:** 2019-05-29

**Authors:** Silvina Ribeiro-Samy, Marta I. Oliveira, Thais Pereira-Veiga, Laura Muinelo-Romay, Sandra Carvalho, João Gaspar, Paulo P. Freitas, Rafael López-López, Clotilde Costa, Lorena Diéguez

**Affiliations:** 1 Department of Life Sciences, International Iberian Nanotechnology Laboratory (INL), Avenida Mestre José Veiga s/n, 4715-330 Braga, Portugal; 2 Roche-CHUS Joint Unit, Oncomet, Health Research Institute of Santiago (IDIS), Complejo Hospitalario de Santiago de Compostela, Trav. Choupana s/n, 15706 Santiago de Compostela, Spain; 3 Liquid Biopsy Analysis Unit, Oncomet, Health Research Institute of Santiago (IDIS), Complejo Hospitalario de Santiago de Compostela, Trav. Choupana s/n, 15706 Santiago de Compostela, Spain; 4 CIBERONC, Centro de Investigación Biomédica en Red Cáncer, Madrid, Spain; 5 Department of Micro and Nanofabrication, International Iberian Nanotechnology Laboratory (INL), Avenida Mestre José Veiga s/n, 4715-330 Braga, Portugal; 6 Department of Nanoelectronics Engineering, International Iberian Nanotechnology Laboratory (INL), Avenida Mestre José Veiga s/n, 4715-330 Braga, Portugal

**Keywords:** Cancer, Biomedical engineering

## Abstract

Liquid biopsy offers unique opportunities for low invasive diagnosis, real-time patient monitoring and treatment selection. The phenotypic and molecular profile of circulating tumor cells (CTCs) can provide key information about the biology of tumor cells, contributing to personalized therapy. CTC isolation is still challenging, mainly due to their heterogeneity and rarity. To overcome this limitation, a microfluidic chip for label-free isolation of CTCs from peripheral blood was developed. This device, the CROSS chip, captures CTCs based on their size and deformability with an efficiency of 70%. Using 2 chips, 7.5 ml of whole blood are processed in 47 minutes with high purity, as compared to similar technologies and assessed by *in situ* immunofluorescence. The CROSS chip performance was compared to the CellSearch system in a set of metastatic colorectal cancer patients, resulting in higher capture of DAPI+/CK+/CD45− CTCs in all individuals tested. Importantly, CTC enumeration by CROSS chip enabled stratification of patients with different prognosis. Lastly, cells isolated in the CROSS chip were lysed and further subjected to molecular characterization by droplet digital PCR, which revealed a mutation in the *APC* gene for most patient samples analyzed, confirming their colorectal origin and the versatility of the technology for downstream applications.

## Introduction

In the last decade the field of liquid biopsies has evolved exponentially^1^. The ability to isolate, detect and analyze tumor-derived material in a minimally invasive way constitutes an exciting and unique opportunity in oncology to investigate tumor dynamics and progression with time^1^. Circulating tumor cells (CTCs) are shed from the primary tumor into peripheral blood and have the capacity to invade other organs, causing metastasis^2^. In fact, CTC enumeration has been correlated with disease progression-free and overall survival in various metastatic solid tumors^3–5^. However, the applicability of CTCs in the clinic has been limited by their scarcity (1–10 CTCs/billion of blood cells) and consequently, by their technically demanding isolation. To date, several different technologies have been developed to capture CTCs^6^, mainly based on either physical or biological properties^7,8^, such as size^9^ or antigen expression^10^. Nevertheless, only the CellSearch system is currently approved by the Food and Drug Administration (FDA) for the use of CTC enumeration in a clinical setting^11,12^. It has been validated as a companion diagnostic tool for prognostic evaluation and therapeutic-response monitoring in patients with metastatic colorectal^3^, breast^12^ and prostate cancer^13^. For metastatic colorectal cancer (CRC), a cut off of ≥3 CTCs in 7.5 mL of peripheral blood has been defined, correlating to an unfavourable prognosis^14^. Despite the clinical utility and validation of CellSearch, its CTC enrichment methodology causes some cell loss, associated to its sample preparation process. Moreover, CellSearch selects solely CTCs expressing the epithelial cell adhesion molecule (EpCAM), likely neglecting other clinically relevant CTC phenotypes. These include mesenchymal and stem cell-like tumor cells that display low levels or totally lack EpCAM expression^15^. In fact, the CTC plasticity has been well documented and attributed to a developmental program designated epithelial-to-mesenchymal transition (EMT), already linked to poorer prognosis and chemoresistance^16–18^. Consequently, by capturing exclusively EpCAM+ cells, the CellSearch system only detects CTCs in up to 60% of the metastatic cancer patients^19^.

Other techniques based on positive enrichment, using anti-EpCAM antibodies^20–23^ also suffer from this limitation, despite showing high capture efficiency of epithelial CTCs. On the other hand, negative-enrichment strategies targeting unwanted blood cells^8^ overcome this biological constrain, but tend to display lower purity yields, hindering downstream analyses^24^. Furthermore, immune-based isolation approaches (positive or negative) require relatively long incubation times (1–8 ml/h) to allow for antigen-antibody interaction^20,25^. Alternatively, label-free approaches based on size exclusion mechanisms have been suggested^26,27^, but sample loss, leukocyte contamination or clogging are frequently a problem^28,29^. The enrichment capacity of these systems is limited to CTCs that display a large diameter in comparison with white blood cells (WBCs), hence losing the smaller CTC population. Diverse 2D and 3D microfilters have been designed^30–34^ and even combined with blood pre-processing using Ficoll^35^ or beads^36^, with higher throughput than in positive immune-selection systems^6^. However, the purity of isolated cells remains the major obstacle of filtration-based methods.

Microfluidics has emerged as a low cost alternative to traditional cell isolation technologies, demonstrating superior sensitivity and enhanced cell recovery^20,37^. Furthermore, it reduces costs by requiring small sample and reagent volumes, and avoids sample-processing steps, lowering cell loss^38,39^. More importantly, all these features can be combined with high-throughput processing, portability and automation, greatly relevant in clinical settings. Microfluidic enrichment of CTCs can be implemented through positive^20,23,40^ or negative-immune selection^25,41^. Yet, as described using benchtop methodologies, the former depends on specific antigen expression and prior knowledge of CTC biomarkers, whereas the latter bypasses CTC heterogeneity but suffers from low throughput. Numerous positive selection microfluidic chips with various geometries and architectures, have been demonstrated to exhibit over 90% of capture efficiency, but only after tedious sample pre-processing steps^6^.

The distinct physical characteristics between CTCs and blood cells, such as size, deformability, density and electrical properties, have also been explored to successfully isolate and enrich CTCs using microfluidic chips. These label-free approaches offer faster processing of high volume samples and do not bias the cell selection, while reducing even further reagent-associated costs. Recent technology advancements include the use of hydrodynamic forces, inertial focusing, acoustic waves or dielectrophoresis to separate different cell populations based on their size^6,42–44^.

In this report, we present the development and pre-clinical validation of a high-throughput microfluidic cell filter, able to efficiently and rapidly isolate CTCs with high purity, directly from unprocessed whole blood of metastatic CRC patients, using a label-free approach. This system allows the isolation of CTCs based on their size and deformability, and also the *in situ* phenotypical characterization of trapped cells, together with their downstream molecular analysis. In addition, we compared the performance of our device against the gold standard CellSearch system, showing higher sensitivity and suggesting a new cut-off for patient stratification.

## Results

### CROSS chip performance in spiked samples

**Figure 1:**
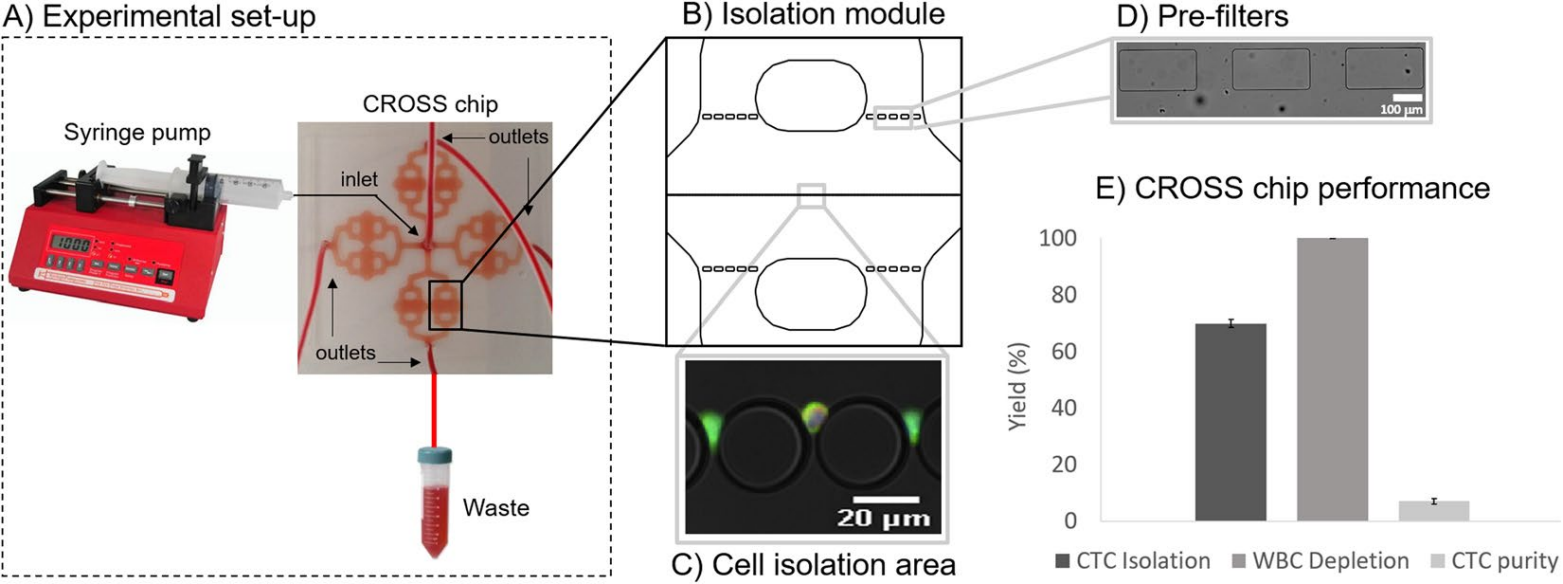
Experimental set-up for CTC isolation using the CROSS chip (**A**). Each chip displays 4 modules containing sets of pre-filters and cell isolation filters (**B**). Across the middle section of each module, a single row of 25 μm anisotropic micropillars spaced 5 μm constitutes the cell filtering area (**C**). The pre-filters present 120 μm gaps (**D**). After optimization in spiked samples, the device shows a CTC isolation efficiency of 70%, WBC depletion capacity of 99.99%, and overall CTC purity of 7.2% (**E**).

Aiming at isolating all CTCs directly from unprocessed blood samples, a label-free microfluidic system for cell capture based on their size and deformability, the CROSS chip, was developed (Fig. 1A–D). The performance of the CROSS chip was investigated using SW480 colorectal cancer cells spiked in whole blood from healthy donors. To achieve the best isolation efficiency in whole blood samples, while maintaining the lowest level of false positives, the flow rate was optimized at 80 μl/min. Notably, the device is able to isolate in average 70% of spiked SW480 colorectal cancer cells, while depleting greatly the WBC population (99.99%), hence maintaining a very high purity (7.2%) (Fig. 1E). This strategy allows fast sample processing, i.e., 7.5 ml of whole blood are processed in 47 min using 2 CROSS chips simultaneously, avoiding potential sample loss and tedious sample preparation procedures.

### Comparative analysis: Isolation of CTCs by CROSS chip versus CellSearch

**Figure 2:**
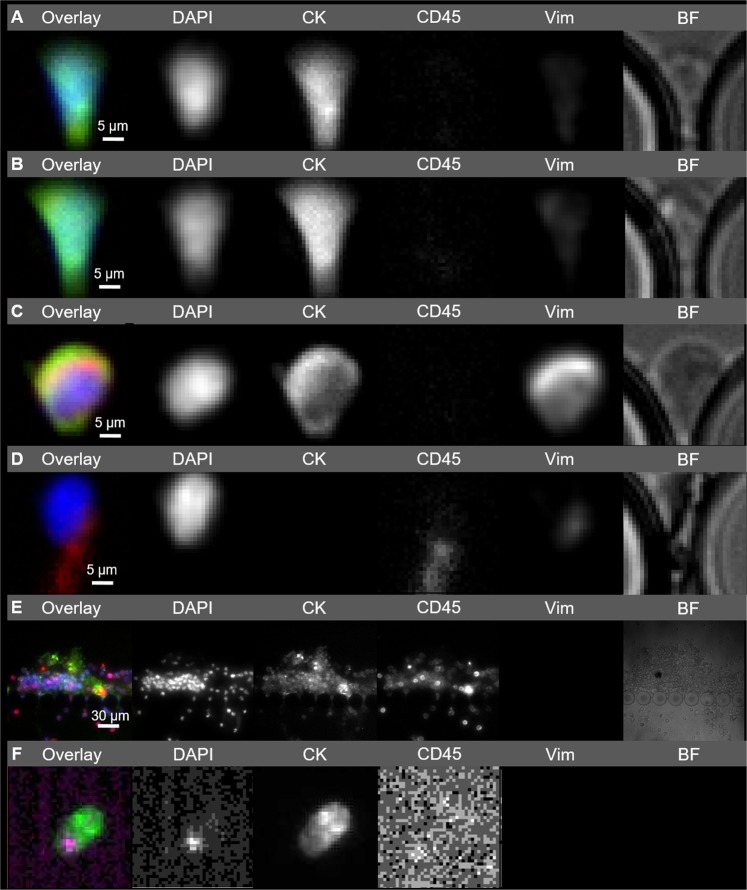
Microscopy images showing cells retained at a central region of the CROSS chip (**A–E**) and identified by CellSearch (**F**). Isolated cells trapped between pillars of the CROSS chip were stained with the following antibodies: anti-pan Cytokeratin-FITC, anti-CD45-Cy5 and anti-Vimentin-eFluor 570, and the nuclear marker DAPI (**A**–**E**). In the case of CellSearch, staining was done with anti-Cytokeratins 8, 9, 18-PE, anti-CD45-APC and DAPI (**F**). Overlay of the fluorescence microscopy images is shown in color (**A**–**F**). Different cell populations with distinct expression profiles can be observed: Epithelial CTCs (**A,B**), EMT/MET CTCs (**C**), WBCs (**D**), and CTC clusters (**E**).

**Figure 3:**
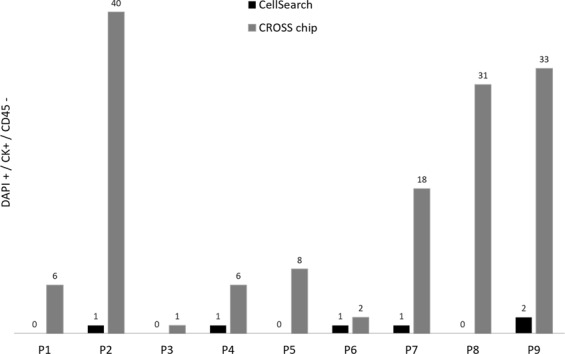
Comparative bar chart demonstrating the enumeration of DAPI+/CK+/CD45− cells (CTCs) using the CellSearch system *versus* the CROSS chip for all nine patient’s samples analyzed in this study.

Considering the good performance of the CROSS chip in spiking experiments, we next moved to its pre-clinical testing. 7.5 ml blood samples from metastatic CRC patients were collected, split in half, loaded in two syringes, and run simultaneously in two CROSS chips. In parallel, another set of 7.5 ml blood samples from the same individuals were collected simultaneously and subjected to CellSearch test. Immunofluorescence staining was used to identify captured CTCs in the CROSS chips, by detecting nucleated, morphologically intact DAPI+/CK+/CD45− cells. Importantly, cells positive for Vimentin and negative for CD45, as well as CK+/CD45− cell clusters were also observed retained in the CROSS device, but not considered for CTC enumeration (Fig. 2). Of note, 7 out of 9 patient samples analyzed showed ≥3 CTCs/7.5 ml of whole blood (mean value = 20.28 ± 14.3) by the CROSS chip. In contrast, none of the patients scored ≥3 CTCs/7.5 ml of whole blood by CellSearch (Fig. 3). No CTCs were detected in the blood of two healthy donors using the CROSS chip.

### Detection of APC mutations in CTCs isolated with the CROSS chip, by ddPCR

**Figure 4:**
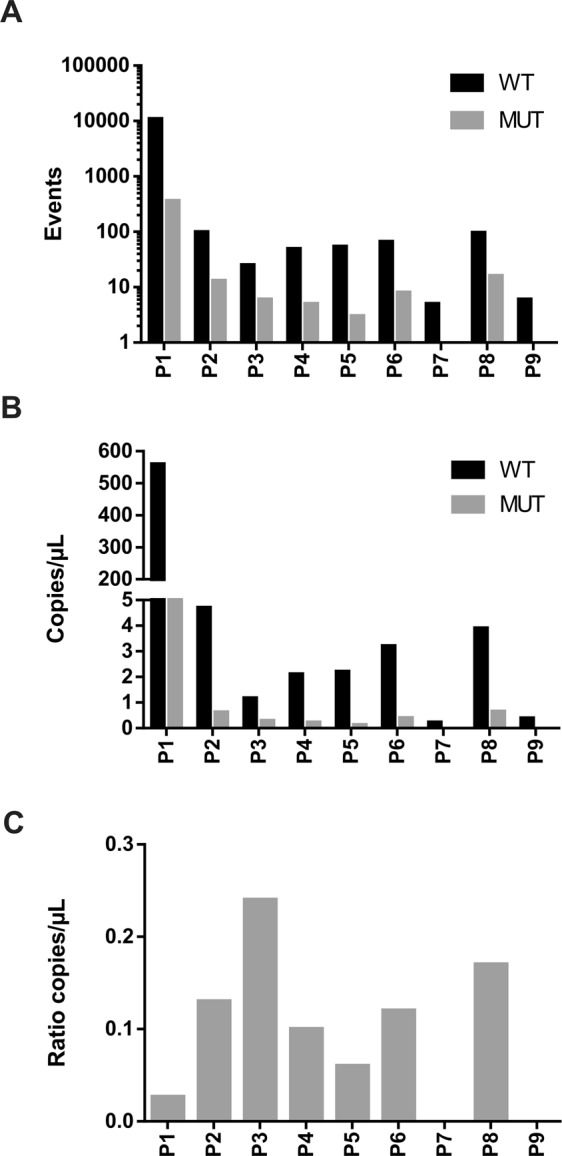
ddPCR analysis for *APC* mutation c.4348C > T for the 9 analyzed patients. (**A**) Number of positive events from QuantaSoft Version 1.7.4.0917 for wild type (Wt) and Mutant (Mut) *APC* gene. (**B**) Copies/µL for Mut and Wt *APC* gene. (**C**) Ratio of copies /µL Mut to Wt, mean: 0.09 ± 0.08. Patients codes P7 and P9 did not present *APC* mutation.

In order to evaluate the origin of the cells isolated using the CROSS chip, CTCs were screened for the most common DNA mutation of the *APC* gene (c.4348C > T), which is highly frequent in CRC patients. Due to the limited amount of starting genetic material available, this analysis was performed by ddPCR. This *APC* mutation was found in 7 out of the 9 patients analyzed, which confirmed the tumor origin of the cells isolated by the CROSS chip (Fig. 4).

### Clinical data correlation and overall survival

**Figure 5:**
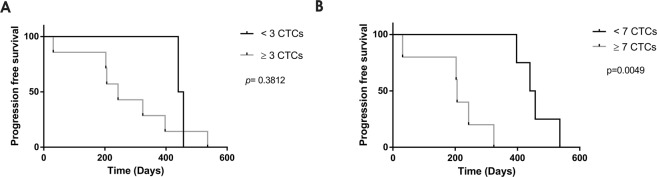
Kaplan–Meier plot of overall survival based on CTCs isolation with CROSS chip. (**A**) Cut off ≥3 CTCs. Median survival <3 CTCs (448.5 days); ≥3 CTCs (243 days). (**B**) Cut off ≥7 CTCs. Median survival <7 CTCs (448.5 days); ≥7 CTCs (206 days).

The number of CTCs enumerated by the CellSearch test was less than 3 CTCs/7.5 ml of whole blood for all samples analyzed (Fig. 3), i.e, below the established cut off for CRC using the CellSearch technology. Considering these data, patients could not be divided in different prognostic groups and all were classified as having good prognosis. However, with the CROSS chip, the CTC number obtained was higher in every patient and thus a possible correlation between CTC enumeration and disease prognosis was investigated. Patients were grouped in good or bad prognosis according to the number of isolated CTCs by the microfluidic device and using the cut-off value defined by CellSearch (<or ≥3 CTCs/7.5 ml of whole blood respectively). As illustrated in Fig. 5A, and according to a Kaplan Meier analysis with a 95% CI, a clear trend for shorter overall survival was observed for patients with ≥3 CTCs/7.5 ml of whole blood than those with <3 CTCs/7.5 mL blood, although not statistically significant (p = 0.3812). Remarkably, defining an alternative cut off of ≥7 CTCs/7.5 ml of whole blood, the CROSS chip is able to discriminate patients with good prognosis from those facing an unfavorable outcome (CTCs ≥ 7) (p = 0.0049), with a greater survival of 242 days (Fig. 5B).

## Discussion

The molecular characterization of a tumor, typically performed on a tissue biopsy, can help therapeutic reasoning and significantly impact the disease outcome. However, tumor heterogeneity and fast evolving dynamics can lead to genetic alterations, making clinical decisions based on historical biopsy data suboptimal^45^. Additionally, tissue biopsies are invasive and not always available, in contrast to CTC-based liquid biopsies. Unraveling the molecular profile of CTCs can provide key information into the biology of the tumor cells and thus improve personalized therapy^2^. However, the use of CTCs as a clinical biomarker is still on hold, given their extremely low frequency and great plasticity^46,47^, making CTC isolation technically challenging. Numerous approaches have been developed to capture these rare cells^2,6,42^, but with disparate results mostly due to ambiguous CTC classification criteria and lack of standard sample preparation protocols^11^. In this work, the CROSS microfluidic filter was developed aiming at rapid and unbiased isolation of unfixed CTCs with high efficiency and purity. This system was tested in a clinical setting and compared to CellSearch using a panel of metastatic CRC patients. Lastly, cells isolated using the CROSS device were screened for the presence of a specific mutation of the *APC* gene, highly frequent in CRC patients, to confirm their malignant origin.

To assess the performance of the CROSS chip, spiking experiments were carried out, demonstrating a high capture efficiency (70%) and purity (7.2%), when using whole blood, in contrast to several size-based technologies that display efficiencies of 40–60%^48–50^. Improved recoveries (up to 95%) have been reported by other label-free methodologies, but using pre-fixed cells^51^, long processing times^52,53^, RBC-depleted^54,55^ or diluted blood^56^ which favor sample loss. The Vortex chip, currently commercially available (Vortex Biosciences), demonstrated high purity (57–94%) but low capture efficiency (up to 37%) of spiked MCF7 breast cancer cells when using diluted blood^57,58^. On the other hand, the ApoStream (ApoCell) was reported to capture up to 75% of spiked renal tumor cells SKOV3 with only 1% purity in processed blood^59^. Warkiani and colleagues showed recoveries of spiked cells around 85% and high depletion of WBCs (99%) for lysed blood samples processed in a curvilinear microchannel chip^60^, now commercialized as the ClearCell FX system (Clearbridge Biomedics). Still, a wide variability in CTC purity was also observed (0.1–86%)^54^. Other commercial microfiltration systems have been reported, such as ScreenCell (ScreenCell)^27^ and CellSieve^TM^ (Creatv MicroTech)^34^ which offer higher throughput (>5 ml/min)^42^ than the CROSS chip. Yet, cell damage, low CTC recoveries and filter clogging are major concerns of mechanical filtration devices given their high flow rate and filtration pressure^28,30^. Notably, all systems described above require sample pre-processing and struggle to discriminate CTCs from WBCs of larger size, often rendering poor isolation of small CTCs or large amount of WBC contamination^61^. To solve this issue, cell deformability can be further explored to discriminate CTCs from similarly sized leukocytes. In this context, capture efficiency rates of 62.5% or higher (>80%) together with minimal leukocyte contamination, have been shown with an optimization of the Parsortix^TM^ (ANGLE) platform^48^ or the Cellsee^TM^ (Celsee Diagnostics) device^51^, using diluted or pre-fixed blood samples spiked with prostate or breast cancer cells, respectively. When using Parsortix and whole blood, capture efficiencies reached up to 70%, similar to that obtained in this work, but only for large size cells (>20 µm)^62^. Instead, with the T-24 bladder cancer cell line (average cell diameter of 18 µm) cell retention dropped to 42%^62^. Moreover, Parsortix, which has a size-restricted separation gap of 10 µm, was limited to running 4 ml of whole blood with a relatively slow sample processing speed^62^, in contrast to the 5 µm-gap of the CROSS chip that processes 7.5 ml in just 47 min. Further systems have shown good ability at isolating CTCs from whole blood, as reported using the Labyrinth chip, which in a double run isolates 91% of CTCs while retaining 663 WBCs per ml analyzed^63^.

Our geometry, combined with our surface treatment, makes for large aspect ratio slippery filters that favor CTC entrapment, while even large white blood cells can be eliminated, reaching a compromise between efficiency, speed and purity. This is due to our anisotropic 5 μm wide/20 μm high filters that, while having smaller width than other filtration-based systems, allow the cells to deform in the vertical axis and squeeze through, retaining only cells which nucleus cannot deform, favoring the trapping of larger cells with high nucleus to cytoplasm ratio. Having just one filtering row prevents air bubble formation inside the device and pre-filters layout avoids clogging that usually takes place when processing whole blood. Although cytomorphological differences in cultured cancer cells and patient derived-CTCs have been acknowledged for prostate cancer^64^, with CTCs being considerably smaller, others have shown that the SW480 cells used in this study are appropriate models to investigate size-based CTCs enrichment systems, as their median diameter (11–13 μm) is very similar to that of colorectal CTCs (11 μm, as found by CellSearch)^65^. Additionally, the filter size of the CROSS chip is smaller (5 μm) than most systems previously reported^33^, including the commercially available ISET (Rarecells; 8 μm)^26^, ScreenCell (ScreenCell; 6.5 or 7.5 μm)^27^ and SmartBiopsy^TM^ (CytoGen; 6.5 × 6.5 μm^2^ square pore)^66^. Hence, it has the potential to retain even smaller/more deformable CTCs, as is the case of mesenchymal and stem-cell-like CTCs^67^. This is in agreement with a recent report that uses a similar architecture for selective CTC isolation and reports on the presence of circulating cancer stem cells^68^.

Preclinical validation of our system was next pursued through a comparative blind study between the CROSS chip and CellSearch test, the gold standard for CTCs enumeration in clinical settings. Importantly, whole blood samples were introduced and filtered directly in the CROSS chip, avoiding sample pre-processing steps performed by numerous CTC isolation techniques including CellSearch^6,51,69^. Notably, using 2 CROSS chips we were able to rapidly (in 47 minutes) process whole blood samples from a set of metastatic CRC patients, and capture a higher number of CTCs than CellSearch in all individuals analyzed, which further reinforces the clinical potential of this system. Immunofluorescence staining of trapped cells corroborated a high capture efficiency and purity of CTCs. The total number of CTCs identified in the CROSS chips was further verified and validated by a technical expert routinely involved in the analysis of CellSearch data. Of the nine patient samples analyzed using CellSearch, all patients were classified as having good prognosis, since the CTC count was below the cut off. Nonetheless, four of the nine samples (44%) had CTCs only detectable by the CROSS chip. As for the other five samples, a great discrepancy was observed in CTC enumeration, with CellSearch reporting 1–2 CTCs and the CROSS chip ranging from 2–40 CTCs (average 19.8 CTCs). These results suggest that the isolation of epithelial CTCs using the CROSS chip is more efficient (*p* = 0.0039) and sensitive than CellSearch. Interestingly, Vimentin+/CD45− cells were also found retained in the CROSS device, indicating entrapment of not just epithelial-like CTCs but also cells with different phenotypes. When accounted for, this will increase even further the number of isolated CTCs and provide additional information of the disease. In fact, a recent study using Parsortix described the isolation of mesenchymal-like prostate CTCs, whose number correlated with worse prognosis^70^. Other microfluidic systems, such as the Vortex HT chip^49^, Parsortix^48^ and Labyrinth^63^ have also reported the capture of heterogeneous CTC subpopulations expressing epithelial, mesenchymal, EMT and/or cancer stem cell markers. The capacity of the CROSS device to isolate not only single CTCs but also CTC clusters, similarly to other systems such as Parsortix^48^ and ClearCell FX^60^, holds great potential as the later have been correlated with higher invasive capacity^71^.

In the scope of the comparative study herein presented, considering the established cut off for bad prognosis in CRC used by the CellSearch technology – i.e. ≥3 CTCs/7.5 ml of whole blood –, the results obtained by CellSearch were negative for all the patients analyzed. In contrast, due to the higher sensitivity, the results obtained with the CROSS chip suggest a new cut off (≥7 CTCs/7.5 ml of whole blood) that stratifies the patients in 2 very well defined populations with overall survival differences higher than 200 days. Yet, further studies on larger cohorts of patients are required to clarify the clinical relevance of this method for CRC monitoring and characterization.

Following CTC isolation, it is of outmost importance to characterize the isolated cells and confirm their tumor origin. Furthermore, this molecular and phenotypic characterization would provide clinically relevant information and be of great utility for therapeutic reasoning. Importantly, the CROSS chip allows downstream molecular analysis of the trapped cells (ddPCR, qPCR, etc), with increased sensitivity given the sample purity, as compared to other technologies^6^. To confirm the malignant origin of the cells isolated by the CROSS chip, the mutational status of *APC*, a tumor suppressor gene that regulates cell cycle and WNT signaling, was evaluated. This is the first time that APC status is described in CTCs isolated by a microfluidic system. For that, we selected a somatic non-sense mutation with high frequency of mutation among patients population. *APC* is the most frequently mutated gene in sporadic CRC, affecting up to 60% of CRC patients^72–75^. Moreover, a strong association between mutations in *APC* and other genes such as *KRAS* or *BRAF* and colon cancer initiation has in fact been established^69,76–79^. In addition, the *APC* gene has been linked to tumor initiation and the frequency of mutation is maintained by the passage of benign to malignant tumors^80^. Some authors suggested that the *APC* mutation has a relevant role in providing a selective advantage, through the activation of the Wnt signal transduction pathway and the chromosomal instability in the tumor cell^81^. Notably, *APC* mutations were detected in CROSS chip-isolated CTCs from 7 out of 9 patients, by ddPCR, even using DNA amounts as low as 0,065 ng/µl. Indeed, ddPCR technique has demonstrated superior sensitivity to detect clinically relevant mutations at very low concentration in liquid biopsies from patients with different malignancies^82,83^. Thus, our findings are in agreement with the overall frequency of *APC* mutation in CRC^84^. Nevertheless, false-negative results cannot be ruled out due to the low amount of starting DNA. A recent work confirmed that in all CRC patients analyzed, the mutational status of *APC* in both CTCs and the primary tumor matched, with 60% concordance^85^. However, to the best of our knowledge this is the first study reporting the analysis of *APC* alterations by ddPCR in CTCs isolated from CRC patients. In a similar study, *APC* mutations were investigated in circulating DNA using the BEAMing technology and were detected in >60% of CRC patients^86^. On an additional note, CRC-derived CTCs isolated by the ScreenCell size-based device have also been screened for mutations in the *KRAS* gene using ddPCR, which were observed in 57% of the cases^87^. In fact, ddPCR is commonly used for ctDNA analysis in oncology and, for example, *KRAS* is analyzed in the clinical routine as it conditions treatment selection for the patients who present *KRAS* mutation.

In summary, although several CTC isolation systems have been described and a fraction even reached commercialization as automated platforms^42^, it should be considered that validation with clinical samples has not always been performed^88,89^. The blood of a cancer patient shows different features compared to that of a healthy donor regarding density or clotting, influencing cell isolation performance of the technologies under investigation. Furthermore, of those studies including patient samples, not all performed a comparison with the only FDA-approved technology CellSearch^66^, crucial to provide an estimated number of captured CTC for each sample, as a positive control. The CROSS chip described herein displayed higher sensitivity than the gold standard, without the need of any sample pre-processing, while allowing downstream molecular analysis, key in a clinical setting. The versatility of this low cost device is further demonstrated by its ability to process blood straight after drawing or more than 24 h post collection.

Besides allowing phenotypic and molecular characterization of captured cells, further advantages of the CROSS chip include easy cell recovery in a very small volume (the internal volume of the system is below 100 μl), by simply inverting the flow and without inducing significant cell damage, particularly important for cell culturing and further downstream applications. This particularity, can enhance the possibilities to establish CTC cultures, which has been to date challenging due mainly to low cell density^90^. Also, the CROSS chip can be used for the study of other cancer types, particularly in diseases where the scarcity of epithelial-like CTCs hinders the applicability of CellSearch. Moreover, the simple setup and protocol used, allows for the deployment of this system in any research or pathology laboratory without the need of any special equipment, and only requiring a syringe pump for CTC isolation.

The CROSS chip has shown limitations in volume capacity but, due to the inner multiplex capacity of the microfluidic systems, it is possible to redesign the system and increase the surface area of the device to analyze higher volumes of whole blood in the same or even less time, having undeniable potential for early cancer diagnosis. Also, further developments will integrate the design in an all-in-one system just comprising a single outlet in a smaller glass slide to facilitate image acquisition. Even though recent studies have shown microfluidic chips able to isolate CTCs with outstanding efficiency and purity from unprocessed blood samples^63^, our latest tests have indicated that the analysis of fresh samples renders higher isolation yields and better quality of the samples, as expected^91^.

From this work we can conclude that the CROSS chip is able to rapidly isolate unfixed CTCs from whole blood with high efficiency and purity, while enabling CTC recovery. Importantly, it shows higher sensitivity than CellSearch when isolating CTCs from metastatic CRC patients, capturing a higher number of cells, even in samples considered negative by the gold standard. The findings obtained suggest that the CROSS chip may allow a better discrimination of patients with poorer prognosis, highlighting its potential as a powerful tool for liquid biopsy studies in CRC and other types of cancer. Moreover, ddPCR confirmed the tumor origin of the isolated cells, and paves the way for further molecular downstream analysis.

## Methods

### Microfluidic device design and fabrication

The CROSS microdevice was designed to split the blood equally in 4 different modules (Fig. 1A). Each module is able to process a maximum of 1 ml of whole blood and contains a set of pre-filters and cell isolation filters (Fig. 1B). Across the middle section of each module, a single row of 700 anisotropic micropillars with diameter 25 μm and spaced 5 μm constitutes the cell filtering area (Fig. 1C). The gap size, geometry and aspect ratio was carefully chosen to allow blood cells to deform and gently flow through, while retaining larger or more rigid cells in the filter. The pre-filters present 120 μm gaps to prevent large clumps or debris from clogging the setup (Fig. 1D). Each microdevice holds an approximate volume of 100 μl. Cells can be retrieved from the system by simply inverting the flow. Surface coating is decisive in both cell isolation and retrieval, as it is crucial to prevent cell attachment to maximize cell purity and recovery.

The microfluidic masters were designed in 2D AutoCAD software (Autodesk, USA) and fabricated on a 200 mm silicon wafer using photolithography and deep reactive ion etching. Briefly, the silicon wafer (P/Boron, <100>, Siegert Wafer, Germany) was rinsed with deionized water, dehydrated at 150 °C and exposed to hexamethyldisilazane (HDMS, Sigma Aldrich, USA) vapour prime to improve the adhesion of the photoresist to the sample. Later, the wafer was spun coated with 2.2 μm of AZP4110 (Microchemicals GmbH, Germany), using a SÜSS MicroTec optical track (SÜSS MicroTec AG, Germany). The pattern was transferred onto the coated wafer using a Direct Write Laser system (DWL 2.0 Heidelberg, Germany) with an Hg laser energy of 95% and focus −50. Following the post bake, the exposed photoresist was developed with AZ400K (Microchemicals GmbH, Germany), and the wafer was rinsed with deionized water and dried. The pattern was then etched with sulfur hexafluoride (SF6, Sigma Aldrich, USA) by Silicon Deep Reactive Ion Etching (STPS Pegasus, United Kingdom), and exposed areas passivated with octafluorocyclobutane (C4F8, Sigma Aldrich, USA). Trench depth was measured in between steps using an optical profilometer (OPM profilometer, Oceon Optics NanoCalc XR) until the desired depth of 20 μm was reached. Residues were stripped using oxygen plasma and the master was characterized by means of Scanning Electron Microscopy (Quanta SEM, FEI, USA). Finally, the wafer was diced into the individual masters using a DAD 3350 Dicing Saw (Disco, Japan) and cleaned with Isopropyl alcohol (IPA, Sigma Aldrich, USA), rinsed with deionized water and dried at 150 °C on a hot plate.

Prior to master replication, the wafer was hydrophobized with a vapor-phase treatment in trichloro(1H,1H,2H,2H-perfluorooctyl)silane (Sigma Aldrich, USA) for 1 h in a desiccator, and cured for another hour at 65 °C. Polydimethylsiloxane (PDMS, Ellsworth Adhesives Iberica, Spain) was mixed at 1:10 ratio, degassed, poured over the master, degassed again and cured at 70 °C for 2 h. After curing, the PDMS was unmolded and inlet and outlets were punched. Finally, clean glass slides and PDMS replicas were treated with oxygen plasma at low power for 15 s and subsequently brought in contact to produce irreversible bonding.

Upon activation with oxygen plasma, the microfluidic devices were connected to a syringe pump and filled with ethanol at 100 μl/min to enhance the wettability; then rinsed with 10 mM Phosphate Buffer Saline (PBS, Sigma Aldrich, USA), and later treated with 1% Pluronic F-127 (Sigma Aldrich, USA) overnight to avoid unspecific attachment of cells onto the channel surface. Considering its cross-shaped design, this CTC isolation device has been designated CROSS chip.

### Cell culture and spiking experiments

The human colorectal cancer cell line SW480 was obtained from ECACC in 2014 and cultured in DMEM (Lonza, Switzerland), supplemented with 10% fetal bovine serum (Invitrogen, USA) and 1% Penicillin/Streptomycin (Invitrogen, USA) at 37 °C with 5% CO_2_. Cells were authenticated and tested for mycoplasma by ECACC and passaged 2–3 times before being aliquoted and stored in liquid nitrogen. No new cell line authentication or mycoplasma testing were performed for thawed cells, passaged for <6 months. When reaching confluence, cells were harvested by incubation in 0.25% Trypsin-EDTA (Invitrogen, USA), washed with PBS and labeled with 12.5 μM Calcein-AM (Sigma Aldrich, USA) according to the manufacturer’s protocol. 100 cells were then spiked in 3.75 mL of whole blood collected from healthy donors and injected at 80 μl/min in the CROSS chip with a syringe pump (New Era Pump Systems, Inc., USA). Trapped cells were rinsed with PBS-2% Bovine Serum Albumin (BSA, Sigma Aldrich, USA), fixed with 4% paraformaldehyde (PFA, Sigma Aldrich, USA) for 15 min at room temperature (RT), stained with 1:10000 DAPI (Sigma Aldrich, USA) for 10 min, and washed again with PBS. Following spiked sample processing, a fluorescence microscopy analysis of the trapped cells was performed using a plan fluor 20x objective (Nikon, Spain) coupled to a fluorescence-adapted inverted Nikon-MA 200 microscope (Nikon, Spain) equipped with a sCMOS camera (Andor Neo scc-01633, Andor Technology Ltd, Ireland). To assess the isolation efficiency of the CROSS chip, the number of Calcein+/DAPI+ cells trapped in the device was compared with the total number of SW480 cells spiked, according to Eq. (1). At the same time, the capacity of the system to deplete the WBC population was calculated by comparing the total number Calcein−/DAPI+ events against the theoretical amount of WBCs in the total volume of blood analyzed (7.5 × 10^6^ WBCs/ml), according to Eq. (2). Finally, the purity of the cell population isolated in the system was determined using Eq. (3). Experiments were done in triplicate.

1 $$CTC\,Isolation\,Efficiency\,( \% )=\frac{Trapped\,CTCs}{Spiked\,cells}\times 100$$

2 $$WBC\,Depletion\,( \% )=\frac{Total\,WBCs\,in\,the\,sample-Trapped\,WBCs}{Total\,WBCs\,in\,the\,sample}\times 100$$

3 $$CTC\,purity\,( \% )=\frac{Trapped\,CTCs}{Total\,number\,of\,cells\,trapped}\times 100$$

### Patient sample collection

Metastatic CRC patients, median age 72.44 years, were recruited at the Medical Oncology Department from the University Hospital Complex of Santiago de Compostela, Spain. Clinicopathological characteristics of the patients are summarized in Table 1. Two 7.5 mL samples of peripheral blood were collected in a CellSave preservative tube (CellSearch, Janssen Diagnosis, USA –now owned by Menarini-Silicon Biosystems, Italy-) and in an EDTA-coated tube, respectively, after informed consent and following the approval and recommendations of the Ethics Committee of Galicia (CAEI2014/126). For control purposes, peripheral blood from healthy donors was collected in EDTA-coated tubes after informed consent. All samples were anonymized and encoded before the analysis.

**Table 1:** Clinicopathological characteristics of patients enrolled in this study.

	n (%)		n (%)
**Age**		**Disease site**	
Median (range)	72.44	Liver	9 (100)
	(67–79)	Lungs nodules	2 (22.22)
		Liver + Lungs	1 (11.11)
		Lymph nodes	1 (11.11)
**Gender**		**Stage**	
Men	6 (66.6)	IV	9 (100)
Women	3 (33.3)		
**Lines of treatment**		**Localization**	
2 lines	6 (66.6)	Colon	4 (44.44)
3 lines	2 (22.2)	Sigma	3 (33.33)
		Recto	2 (22.2)

### Patient sample analysis

CellSearch results analysis was performed by the Liquid Biopsy Analysis Unit of the University Hospital Complex of Santiago de Compostela, Spain, as previously described^92^. Whole blood samples to be analyzed in the CROSS chip were collected in EDTA-coated tubes before or after treatment onset, shipped at RT and processed the day after collection. Each EDTA tube containing 7.5 ml of whole blood was divided in half and 3.75 ml processed in each of two CROSS chips, as in the spiking experiments.

### Immunofluorescence and CTC enumeration in the CROSS chip

Isolated cells from patient samples were permeabilized with 0.25% Triton X-100 solution (Sigma Aldrich, USA) and fluorescently labeled inside the microfluidic device with anti-pan CK-FITC (clone C-11, recognizes human cytokeratins 4,5,6,8,10,13, and 18, Sigma; 1:100 in PBS-2% BSA), anti-Vimentin eFluor 570 (eBioscience, 1:50 in PBS-2%BSA) and anti-CD45-Cy5 (Abcam; 1:25 in PBS-2%BSA) antibodies for 1 h. DAPI (Sigma Aldrich, 1:1000 in PBS-2%BSA) was used as a nuclear marker. Fluorescence microscopy analysis was performed using the same setup as described in the cell culture and spiking experiments sub-section. Only DAPI+/CK+/CD45− cells were considered for CTCs enumeration, whereas DAPI+/CK−/CD45+ represented leukocytes. CTC quantification was performed adding the number of cells isolated in the 2 CROSS chips used for each analysis, with blind scoring and by 3 different experienced examiners. The ability of the CROSS device to isolate epithelial-mesenchymal (EMT) or mesenchymal-epithelial (MET) transitioning CTCs was evaluated by confirming the presence of DAPI+/Vim+/CD45− cells.

### DNA extraction and ddPCR analysis

Extraction of genomic DNA from cells retained in the microfluidic devices was performed using AllPrep DNA/RNA Mini Kit (Qiagen, USA). Firstly, cells were lysed upon injection of a lysis buffer (Buffer RLT) at 80 µl/min followed by 5 min incubation and a second injection of the same buffer at 250 µL/min to collect all cell content. Subsequent steps were performed according to the manufacturer’s recommendations. Quantification of the extracted genomic DNA was performed with the Quantifluor ONE dsDNA System using Quantus Fluorometer (Promega, USA).

Absolute quantification of *APC* transcript was performed by ddPCR analysis (QX200™ Droplet Digital™ PCR System, Bio-Rad, USA) at the Universitat Autònoma de Barcelona (UAB) Scientific Technical Services (Barcelona, Spain). Prior to quantification, samples were digested (HaeIII, Sigma-Aldrich, USA) and preamplified (Sso Advanced Preamp Supermix, Bio-Rad, USA). ddPCR experiments were performed using probes dHsaCP2500509 and dHsaCP2500508 for *APC*. The droplets were quantified using the Bio-Rad Quantisoft software. Two replicates per sample were performed.

### Statistical analysis

Statistical analysis was performed using GraphPad Prism software, version 6.01 (GraphPad Software, USA). The Wilcoxon signed rank test (95% confidence intervals) was used to compare CTC enumeration using CellSearch test versus the CROSS filter from the same metastatic patient, whereas Kaplan Meier method was used for survival analysis from time of sample collection. Findings of p < 0.05 were considered statistically significant.

## Data Availability

The datasets generated during and/or analysed during the current study are not publicly available, being now exclusively licenced to a for-profit company, but are available from the corresponding author on reasonable request.
